# Evaluation of Simultaneous Nutrient and COD Removal with Polyhydroxybutyrate (PHB) Accumulation Using Mixed Microbial Consortia under Anoxic Condition and Their Bioinformatics Analysis

**DOI:** 10.1371/journal.pone.0116230

**Published:** 2015-02-17

**Authors:** Jyotsnarani Jena, Ravindra Kumar, Anshuman Dixit, Sony Pandey, Trupti Das

**Affiliations:** 1 CSIR-Institute of Minerals and Materials Technology, Bhubaneswar, Odisha, India; 2 Institute of Life Sciences, Bhubaneswar, Bhubaneswar, Odisha, India; Purdue University, UNITED STATES

## Abstract

Simultaneous nitrate-N, phosphate and COD removal was evaluated from synthetic waste water using mixed microbial consortia in an anoxic environment under various initial carbon load (ICL) in a batch scale reactor system. Within 6 hours of incubation, enriched DNPAOs (Denitrifying Polyphosphate Accumulating Microorganisms) were able to remove maximum COD (87%) at 2g/L of ICL whereas maximum nitrate-N (97%) and phosphate (87%) removal along with PHB accumulation (49 mg/L) was achieved at 8 g/L of ICL. Exhaustion of nitrate-N, beyond 6 hours of incubation, had a detrimental effect on COD and phosphate removal rate. Fresh supply of nitrate-N to the reaction medium, beyond 6 hours, helped revive the removal rates of both COD and phosphate. Therefore, it was apparent that in spite of a high carbon load, maximum COD and nutrient removal can be maintained, with adequate nitrate-N availability. Denitrifying condition in the medium was evident from an increasing pH trend. PHB accumulation by the mixed culture was directly proportional to ICL; however the time taken for accumulation at higher ICL was more. Unlike conventional EBPR, PHB depletion did not support phosphate accumulation in this case. The unique aspect of all the batch studies were PHB accumulation was observed along with phosphate uptake and nitrate reduction under anoxic conditions. Bioinformatics analysis followed by pyrosequencing of the mixed culture DNA from the seed sludge revealed the dominance of denitrifying population, such as *Corynebacterium*, *Rhodocyclus and Paraccocus* (*Alphaproteobacteria* and *Betaproteobacteria*). Rarefaction curve indicated complete bacterial population and corresponding number of OTUs through sequence analysis. Chao1 and Shannon index (H’) was used to study the diversity of sampling. “UCI95” and “LCI95” indicated 95% confidence level of upper and lower values of Chao1 for each distance. Values of Chao1 index supported the results of rarefaction curve.

## Introduction

Polyhydroxyalkanoate (PHA), a bio-polymer currently under scrutiny as an alternative to petroleum based plastics, is a metabolic by-product of microorganisms active in wastewater treatment plants (WWTP) operating under high substrate load [1–6]. Wallen and Rohwedder [7] for the very first time reported PHA accumulation in microorganisms performing Enhanced Biological Phosphate Removal (EBPR) in a WWTP. Now it is well known that a conventional EBPR process achieves nutrient removal under a sequential anaerobic-aerobic/anoxic state by a specific group of microorganisms termed as PAOs (Phosphate Accumulating Organisms). Depending on the substrate type, either polyhydoxybutyrate (PHB) or polyhydroxyvalerate (PHV) is synthesized by the microorganisms in the anaerobic phase along with phosphate release due to splitting of the internal polyphosphate pool to produce the required ATP [8]. In the subsequent electron rich aerobic/anoxic phase, stored PHB is utilized to replenish the phosphate pool resulting in overall phosphate removal [8–10]. Therefore, it is obvious that simultaneous phosphate accumulation and PHB recovery is not a feasible option in a conventional EBPR system. PHB recovery from EBPR enriched sludge is a well documented phenomenon, however, most of these studies don’t focus on nutrient removal [11, 12]. Reports on EBPR system achieving PHB accumulation without disturbing nutrient removal are sparse [12, 13].

Apart from operating conditions, a range of diverse micro flora might also contribute to simultaneous PHB accumulation and nutrient removal. Initially the presence of nitrate was believed to have an inhibitory effect on phosphate accumulation by PAOs, as nitrate rich medium was observed to be more suitable for proliferation of the denitrifiers [1]. Later it was observed that under anaerobic/anoxic system phosphate accumulation was being performed by a group of denitrifiers, using the intracellular PHA/PHB as electron donor and nitrate as terminal electron accepter. Subsequently this group of microorganisms was termed as DNPAOs [8]. Schuler and Jenkins [13] and later Zhou et al. [14] reported that by means of glycogen degradation as an alternate energy source, PAOs can accumulate PHA without releasing phosphate. A few other reports also suggest that denitrifying bacteria don’t have to undergo PHB hydrolysis for phosphate uptake [15, 16]. Efficacy of the dynamic EBPR sludge has been validated for PHB recovery under different operational conditions like complete aerobic, aerobic dynamic feeding with variables like HRT, pH, SRT etc [17, 18]. However, there is a dearth of information as to what would be the behaviour of the microorganisms and the reactor performance under complete anoxic conditions with surplus external substrate. Under such a state, when both electron donor and acceptor are available in surplus, the microorganisms might function differently, which in turn might alter the scenario of conventional EBPR.

The anoxic system, if optimized, can find a way to resolve two major environmental challenges of the current era, i.e, industrial/municipal effluent treatment along with the production of a biodegradable compound. A carbon source in anoxic condition has been reported to inhibit phosphate uptake [19, 20]. At a later stage, researchers have determined a specific group of bacteria capable of accumulating phosphate in the presence of both nitrate and acetate [15]. Substrate (carbon) concentration is understood to be analogous with high PHB accumulation, however nutrient release has also been reported under similar conditions [3, 6]. Therefore, it is crucial to optimize the carbon concentrations in the reactor en route for development of a sustainable process that can strike a balance between optimum nutrient removal with simultaneous PHB accumulation.

In the current work, a set of batch experiments has been designed to study the potential of enriched microbial consortia for simultaneous nutrient removal and PHB accumulation under complete anoxic conditions with varying ICL. As a mixed bacterial consortium help maintaining the dynamism of the overall system; therefore the microbial analysis of the seed sludge was conducted using high throughput sequencing techniques, to determine the key players in the process.

## Materials and Methods

### 1. Synthetic waste water

Synthetic wastewater (KNO_3_–1.63 g/L as nitrate source, KH_2_PO_4_–0.043 g/L as phosphate source, MgSO_4_–1.5 g/L, peptone-0.38 g/L), and 0.3mL of nutrient solution (0.15g/L FeCl_3_.6H_2_O, 0.15 g/L H_3_BO_3_, 0.03g/L CuSO_4_.5H_2_O, 0.18g/L KI, 0.12 g/L MnCl_2_.4H_2_O, 0.06 g/L Na_2_MoO_4_.2H_2_O, 0.12 g/L ZnSO_4_.7H_2_O, 0.15g/L CoCl_2_.H_2_O, 10g/L EDTA) was used in all the batch reactors [21]. Synthetic waste water was sterilized prior to the experiment. Sodium acetate was added in different concentrations (2 g/L-ICL1; 4 g/L-ICL2; 6 g/L-ICL3; 8 g/L-ICL4 and 10 g/L-ICL5) in order to vary the ICL and depending on the concentration of carbon, the initial COD/NO_3_ ratio in different batch experiments were 1.75 for ICL-1, 3.7 for ICL-2, 5.3 for ICL-3, 7.1 for ICl-4, 8.9 for ICl-5. Deliberately high COD/NO_3_ ratio was opted to evaluate the reactor performance to achieve simultaneous COD and nutrient removal along with PHB accumulation by the microbial consortia.

### 2. Batch reactor set up

Reactors having 1L of working volume connected with a pH probe and nitrogen purging tube were used as the batch reactors for each set of experiment. 50 mL of activated sludge from a Sequencing Batch Reactor (SBR) performing biological nutrient removal was used as inoculum in each set. After inoculation, the reactors were sealed and aseptic conditions were maintained in order to prevent any contamination during the experimental period.

In the batch reactors nitrogen gas (~99.99% purity) was passed throughout the run time to maintain complete anoxic condition. The initial pH of the medium ranged between 7.2–7.5. Representative samples were drawn for the analysis of various parameters at regular intervals within the experimental run time of 6 h.

To validate the exact effect of nitrate on phosphate and COD removal, one set (ICL2) was repeated with longer experimental period. The reactor was supplemented with 0.5g/L of nitrate solution at the 6^th^ hour of the experiment, i.e. after exhaustion of initial nitrate. Then the reactor performance was monitored for the next 6 hours (depending on the time taken for complete exhaustion of nutrient and the residual COD).

### 3. Operational condition of SBR providing seed sludge for the batch studies

Activated sludge was drawn at the beginning of anoxic phase from the SBR and was inoculated to each set of batch reactor as seed sludge. This was done to maintain the uniformity of microbial population during the batch experiments.

Composition of synthetic wastewater and all the other parameters like initial pH, NO_3_, PO_4_, COD concentrations and time period of the anoxic cycle of SBR was similar to the batch studies excepting the ICL. The SBR was continuously being operated for 180 days in a 12 hour cycle with anoxic (6h) -aerobic (5h)—settle-decant-refill (1h) phases. It was validated for high nutrient removal efficiency during each cycle, which supports the prevailing steady state condition during the period of operation of the SBR (unpublished data). However the potential of microorganisms (in the SBR) for PHB accumulation was only validated with sudan black staining prior to the batch experiments.

### 4. Analytical methods

Collected samples were filtered (0.45 µm pore size), filtrates were analyzed for nitrate-N, phosphate and COD using standard techniques [22]. Volatile Suspended Solid (VSS) measurement was done for each experimental set up. Sudan black staining procedure was used to substantiate the accumulation of PHB granules inside the microbial cells. Extraction and estimation of PHB were performed using standard protocol [3, 23].

### 5. Characterization of PHB

**5.1 Solid state H-NMR analysis.** Chemical analysis of the polymer was done using ^1^H nuclear magnetic resonance (NMR). 1 mg of the polymer was dissolved in 1 mL of CDCL_3_ and the spectra of the standard (commercially available PHB from SIGMA Aldrich) as well as test samples were obtained by using a Jeol FT-NMR (400MHz of the coupling constant).

**5.2 Fourier transforms infrared spectroscopy (FTIR).** The extracted PHB was further analyzed by FTIR using available standards as reference. A PerkinElmer spectrum GX FTIR was used for analysis with a spectral range of 4000 cm^-1^ to 40 cm^-1^ and resolution of 0.15 cm^-1^.

### 6. Microbial community exploration

Microbial community was studied by 16s rDNA analysis. DNA samples extracted from the seed sludge during the beginning of anoxic phase of SBR were analyzed through pyrosequencing technique. This sludge was used in each batch experiment conducted under aseptic conditions. It is quite evident that though there may be a variation in the population size in different batch studies, the overall microbial community is supposed to be the same as in the SBR.

In a separate SBR which was operational under similar conditions as mentioned above; 16S rDNA analysis of the microbial community revealed a similarity in population dynamics with change in operational conditions (Unpublished data). This again suggests that the population dynamics in the batch reactors would have been similar to the dynamics in the seed sludge.

Therefore, batch reactor performance was supported by both chemical and microbial community (16S rDNA) analysis rather than solely depending upon any one technique.

Total DNA was isolated from the seed sludge sample using a DNA isolation kit (Fast DNA Spin Kit, MP Biomedicals) as per the manufacturer’s protocol. Bacterial 16S rDNA genes from the total bacterial DNA were amplified by PCR followed by pyrosequencing at the Research and Testing Laboratory (RTL), Texas, USA.

In the current study, we have used two different software programs, namely Ribosomal database project (RDP) [24] and DECIPHER [25]. The RDP is a collection of various tools to analyze data for microbial population from large sequencing libraries. DECIPHER has extensive capabilities for identification of chimeric sequences. The analysis of sequencing data was done as outlined below.

**6.1 Pre-processing.** Raw reads were treated by pyrosequencing pipeline of RDP modules using default settings. Primers, adaptors and barcodes were trimmed from each read. The sequence reads with poor quality scores were removed from further analysis. Thereafter, DECIPHER was used to filter chimeric sequences. The remaining sequences reads were used for further analysis.

**6.2 Calculation of rarefaction curve and diversity indexes.** The pre-processed sequence reads were aligned by infernal [26] using the bacteria-alignment model (model-9). Complete linkage clustering was performed to assign these reads to the Operational Taxonomic Units (OTUs) at the distance threshold of 0.01, 0.03, 0.05, 0.07 and 0.10. Rarefaction curve and diversity indices (Chao1 richness and Shannon index) were calculated on the basis of these clusters. These steps were done using RDP.

**6.3 Taxonomic classification.** The sequence reads were classified into different taxonomic classes using RDP Classifier tool. It uses a naïve Bayesian classification algorithm for classification of sequences into different taxonomic classes. A bootstrap cutoff of 50% as suggested by RDP developers was applied to assign the sequences to different taxonomic levels.

## Result and Discussion

### 1. Nutrient and COD removal

**Figure 1:**
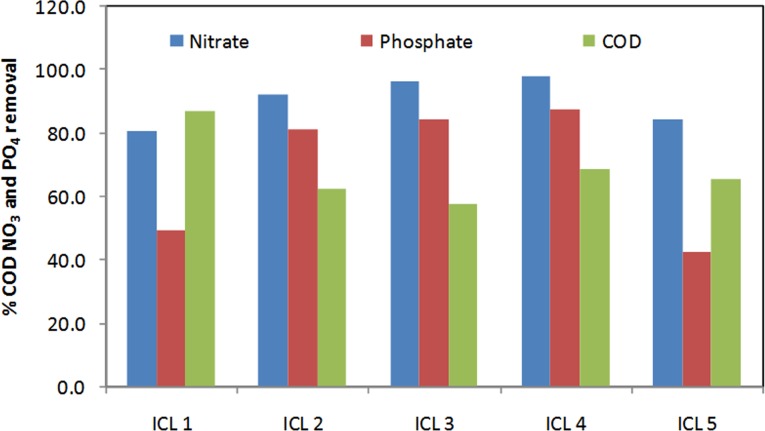
% of removal of phosphate, nitrate-N and COD under different ICL.

**Figure 2:**
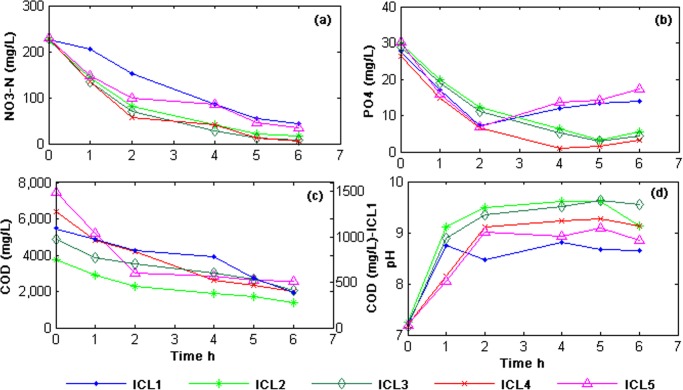
NO_3_ (a), PO_4_ (b), COD(c) removal and pH (d) trend in the batch reactors operated for 6 h.

Nutrient removal efficiency has been evaluated by monitoring the concentration of phosphate and nitrate-N in each set of experiment for a period of 6 h. Beyond 6 hours nutrient removal was depleted as more than 95% of nitrate-N was utilized. Hence the reactor run time was kept limited to 6 h initially. Fig. 1 represents the percentage removal of phosphate, nitrate-N and COD under different ICL. It has been assumed that the COD load in the batch reactor is directly proportional to the ICL [27]. It can be observed in Fig. 2 (a) that variation of ICL had a direct impact on nitrate-N removal rate.

Within 6 hours of incubation nitrate-N removal was significant in ICL-1 (81%), ICL-2 (92.3%), ICL-3 (96.4%), ICL-4 (97.9%) and ICL-5 (84.7%). Low initial carbon load (in ICL-1) might have hindered the nitrate-N utilization rate [28]. ICL-2, 3 and 4 showed a substantial and sequential rise in the nitrate removal rate indicating the importance of an optimal COD/NO_3_ ratio in the medium that supports the growth of the enriched biomass playing a major role in the nutrient removal process. These results are concordant with previous studies [27, 29, 30]. In ICL-5 nitrate removal rate declines in spite of a high COD/NO_3_ ratio, which has been reported earlier to have favoured nitrate reduction [30, 27]. In ICL-5 a rise in carbon concentration also lowers the phosphate/COD ratio to 0.004 in the medium which has been stated [31, 32] to be a favourable condition for the metabolism of other heterotrophic organisms (OHOs) like Glycogen Accumulating Organisms (GAOs) [33–35]. Under similar conditions PAOs are also reported to behave as GAOs [14] and a low phosphate/COD ratio is usually preferred for enrichment of GAOs [31, 36]. GAOs are capable of nitrate reduction and acetate uptake under anoxic condition and are termed as DGAOs (Denitrifying Glycogen Accumulating Organisms), however, they lack the ability to recycle phosphate [37, 38]. Therefore, it is evident that in ICL-5, DNPAO performances were partially affected by other heterotrophs like DGAOs which could have become more active due to suitable growth conditions. This is in agreement with other studies [14, 31, 37]. Microbial analysis of the seed sludge (described in a later section) has revealed the co-existence of DNPAOs and DGAOs in the mixed consortium however, due to the prevailing conditions in ICL-5 the population size of both DNPAOs and DGAOs might have been altered during the course of the experiment.

It was observed that variation in ICL also had a significant impact on the phosphate removal trend (Fig. 1, Fig. 2b). Overall, 81.5%, 82.4% and 87.4% of PO_4_ removal was achieved in ICL-2, 3 and 4 respectively within 6 hours of incubation. Discrepancy in PO_4_ removal in ICL-1 (49.3%), and ICL-5 (42.8%) might be due to scarcity of carbon source in the former and high concentration of carbon that disturbs the growth and maintenance of DNPAOs in the later as described in the earlier section [27, 39]. A clear explanation can be derived by comparing Fig. 2a with Fig. 2b. In ICL-2 and 3, nitrate-N concentration touches the minimum value at 5 hours of incubation, and the PO_4_ concentration goes up (Fig. 2b) which indicates that a decline in nitrate-N concentration in the medium puts stress on the microorganisms and probably deplete the stored polyphosphate for energy; thus releasing inorganic PO_4_ to the medium. Further, it is observed that in ICL-4, PO_4_ release occurs beyond 4 hours of incubation with the subsequent drop in nitrate-N concentration almost during the same time. Interestingly, in ICL-1 and ICL-5, PO_4_ was released into the medium beyond 2 hours of incubation, though during that time nitrate-N concentration was plenty. The carbon load in ICL-1(less than optimal) and ICL-5 (more than optimal) might have triggered a stressful state for DNPAOs leading to an early PO_4_ release by the microorganisms as described earlier. Under the prevailing conditions in ICL-5, DGAOs/OHOs might have brought about a partial disturbance in phosphate utilization by DNPAOs, limiting the phosphate uptake to only 42.8%. These observations are at par with previous results [35, 40].

Highest COD removal was achieved in ICL-1 (87%) followed by ICL-4 (69%), ICL-5 (66%), ICL-2 (63%) and ICL-3 (58%) (Fig. 2c). Initial nitrate-N concentration in ICL-1 was sufficient to facilitate maximum utilization of minutely available COD by the biomass. In ICL-5 considerable COD and nitrate-N removal with phosphate release was observed beyond 2 hours of incubation. Previous studies have also reported the outgrowth of DNPAOs by OHOs under a high COD/NO_3_ ratio as the nitrate load is insufficient to support the process of denitrification by DNPAOs [35, 41]. Therefore, in ICL-5 a high COD/NO_3_ ratio along with a low phosphate/COD ratio supports proliferation of OHOs/DGAOs. In other sets (ICL-2, 3 and 4) a rapid consumption of nitrate-N left the amount of residual COD high. It has been explained later that the addition of extra nitrate-N to the reactor after exhaustion (beyond 6h) brought about a remarkable decrease in the final COD (86% of removal was achieved in ICL-2).

**Figure 3:**
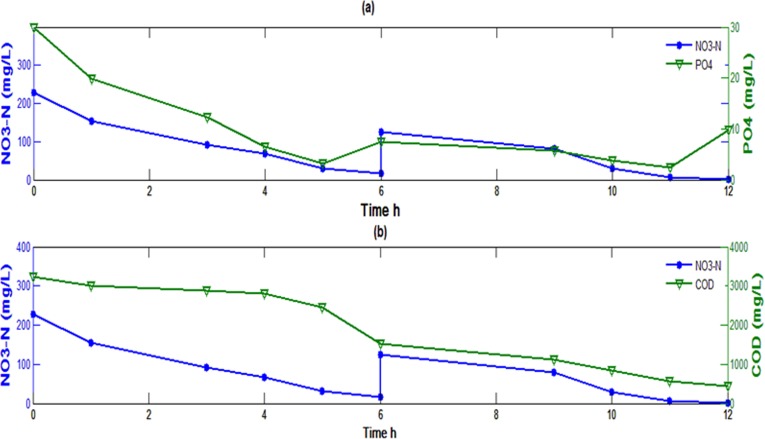
Effect of addition of extra nitrate-N after exhaustion of initial nitrate-N in the reactor on (a) phosphate removal (b) COD removal.

**1.1 Importance of Nitrate-N in an anoxic reactor.** The efficiency of all batch experiments, described in the prior section, was disturbed following nitrate-N exhaustion in the medium. Therefore, it was apparent that nitrate-N was the limiting factor and depletion of nutrient removal rate was directly correlated with availability of electron acceptor [42, 43] i.e. nitrate-N in the present study. Exhaustion in nitrate-N concentration generates stress that probably leads to breakdown of Poly P and release of phosphate to the medium [44]. Further investigations were carried out by supplying extra nitrate-N (110 mg/L) into the anoxic reactor followed by the exhaustion of initial nitrate-N supplied at the beginning of the reaction. It was observed that with addition of extra nitrate-N, to the batch-reactor (ICL-2) in the 7^th^ hour of experiment, more than 90% of PO_4_ (Fig. 3a) and COD removal was achieved (Fig. 3b).

Results of the batch studies indicate that concentration of initial nitrate-N and carbon load are interdependent parameters for achieving ideal nutrient and COD uptake from wastewater. It was also presumed that in spite of a high carbon load, the performance of an anoxic reactor can be maintained at an ideal state (achieving maximum COD and nutrient removal) provided adequate nitrate-N is available for utilization as electron acceptor by the microorganisms. From this set of experiment COD/NO_3_ ratio of 2.3 was found to be optimum for the growth and metabolism of the microbial consortium in the present case to achieve maximum nutrient and COD removal.

### 2. pH trend in the experiment

Irrespective of initial carbon load, pH of the reaction medium showed a rising trend in all the sets due to prevailing denitrifying condition (Fig. 2d) [45]. Increase in pH along with nitrate-N reduction was a virtual indicator of active metabolic trait of DNPAOs in the reactor. The increasing trend of pH was seized after exhaustion of nitrate-N (6h), indicating a decline in the rate of denitrification. Further it can be noticed that the pH trend is alike in ICL-2, ICL-3 and ICL-4 for which nitrate-N removal rate is more or less similar. Whilst in ICL-1 the trend shows an initial increase followed by a gradual downfall beyond 3 h of incubation and in ICL-5 the trend is slightly uneven. It indicates the discrepancies in denitrification rate in both the sets which ultimately results low in nutrient removal.

### 3. Kinetic study

In this study nitrate-N was found to be the limiting factor for nutrient and COD removal.

Therefore Rate of nitrate-N utilization under various ICL can be explained by the following equation:

$$Rate=\frac{-dN}{dt}=K(N)$$

Where N is the nitrate-N and K is the specific reaction rate constant.

Integration of (eq 1) gives the following

$$-\text{ln}\frac{N_{t}}{N_{0}}=Kt$$

Where N_t_ is the concentration of nitrate-N at time t and N_0_ is the initial nitrate-N concentration in the reaction medium.

**Figure 4:**
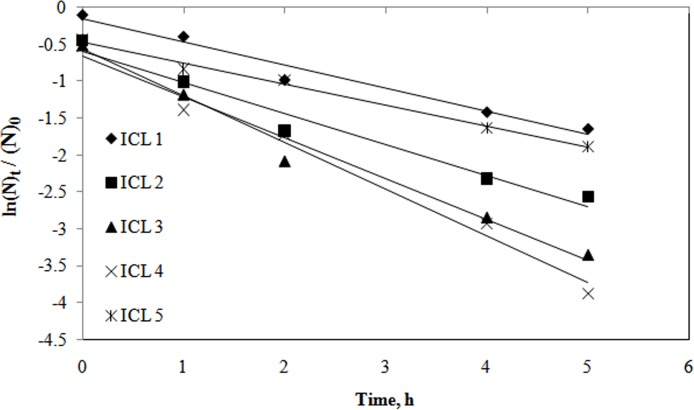
First order plot for nitrate-N utilization under various initial carbon load (ICL-1 to ICL-5).

Considering the reaction to be first order a plot of $\ln\frac{N_{t}}{N_{0}}$ versus time gives a straight line and from the slope k was calculated (Fig. 4). The R-squared value for each line was observed to be >0.9. Hence it was assumed that the nitrate-N removal rate by activated sludge was a first order reaction. Values of ‘k’ are 0.311, 0.419, 0.552, 0.63 and 0.283 for ICL-1, ICL-2, ICL-3, ICL-4 and ICL-5 respectively.

### 4. PHB accumulation

Sudan black staining of biomass confirmed the accumulation of PHB [3]. DNPAOs uptake excess external carbon and store it as PHB in the presence of nitrate-N as luxury PHB uptake [2]. It was further observed that nitrate-N did not have an adverse effect on PHB accumulation in the current study, as was reported earlier [1, 46].

**Figure 5:**
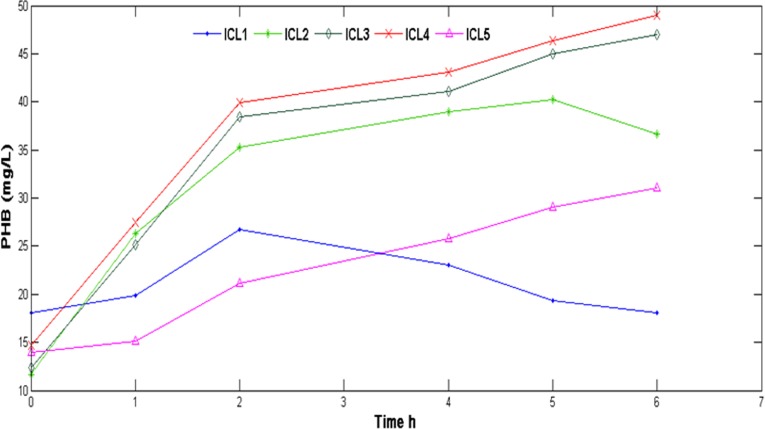
PHB accumulation in different sets with different ICL.

Fig. 5 shows the variability of PHB accumulation in the bacterial cell (mg of PHB accumulated per 1L activated sludge) with respect to different ICL. It can be observed that PHB accumulation was directly proportional to the organic carbon load; with less PHB accumulation at lower carbon load and vice versa (except ICL5). However, time taken for high PHB accumulation (5h in ICL-2 and beyond 5h in ICL-3) increased with an increase in initial carbon concentration [46]. In ICL-5, the PHB accumulation shows an increasing trend but during 6 hours of incubation 30 mg/L of PHB was accumulated which was lower than ICL-3 and 4, where more than 45 mg/L of PHB accumulation occurred during the same time period. As has been described earlier DNPAO population, supposed to accumulate PHA are impaired which could have lead to the discrepancy in PHB accumulation observed in ICL-5.

ICL-1 experienced a slight increase in PHB concentration, due to utilization of ~200mg/L of COD within 2 hours of incubation and a slight fall in concentration was observed beyond that; even though the COD concentration was showing a decreasing trend during the same time. This inconsistency might be caused due to a lower concentration of external carbon. It is obvious that PHB accumulation occurs only when the availability of external carbon source is in excess [47, 48] but interestingly a decline in PHB concentration is also marked with simultaneous release in PO_4_ concentration indicating a stressful environment for DNPAO population. This typical observation further justifies the fact that a part of COD might have been utilized for growth and maintenance of the bacterial consortia. However, the exact reason behind the PHB break down with simultaneous phosphate release beyond 2 hours of incubation could not be addressed in the current study. Could this be a mechanism adapted by the consortia to deal with stress conditions? Further investigation is required to validate such an assumption.

Similarly the slight depletion in the PHB along with phosphate release beyond 5 h of incubation in ICL-2 indicates the development of a stress condition followed by nitrate-N exhaustion as described earlier. In conventional EBPR, hydrolysis of PHB facilitates poly P build up [49, 50] but this hypothesis could not be justified in the current study as PHB depletion was not accompanied by subsequent phosphate uptake from the medium rather phosphate was released beyond 5 h of incubation (Fig. 2). 16s rDNA analysis of the seed sludge is evident of the fact that the most abundant population in the sludge, *Paracocous* does not utilize PHB for Poly P formation [15].

### 5. Characterization of the Polymer

**Figure 6:**
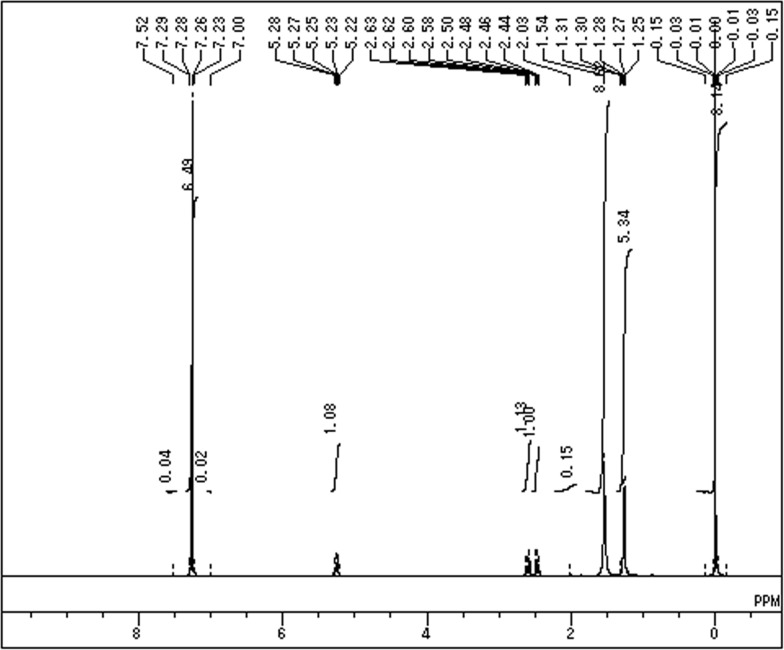
H-NMR spectra of extracted Polymer.

**5.1 H-NMR analysis.** H-NMR analysis was used to determine the structure of the polymer recovered from the sludge. The spectrum confirmed the polymer to be PHB showing a doublet peak at 1.25 ppm alkyl (secondary and tertiary), doublet of quadruplet peaks between 2.4ppm attributed to a methylene group adjacent to asymmetric carbon atom and a single peak at 5.2ppm also attributed to a methylene group (Fig. 6) [51, 52]. A large peak in between 7–7.5ppm might be due to the departed CDCL_3_ and the peak at 0.01 is due to water contamination.

**Figure 7:**
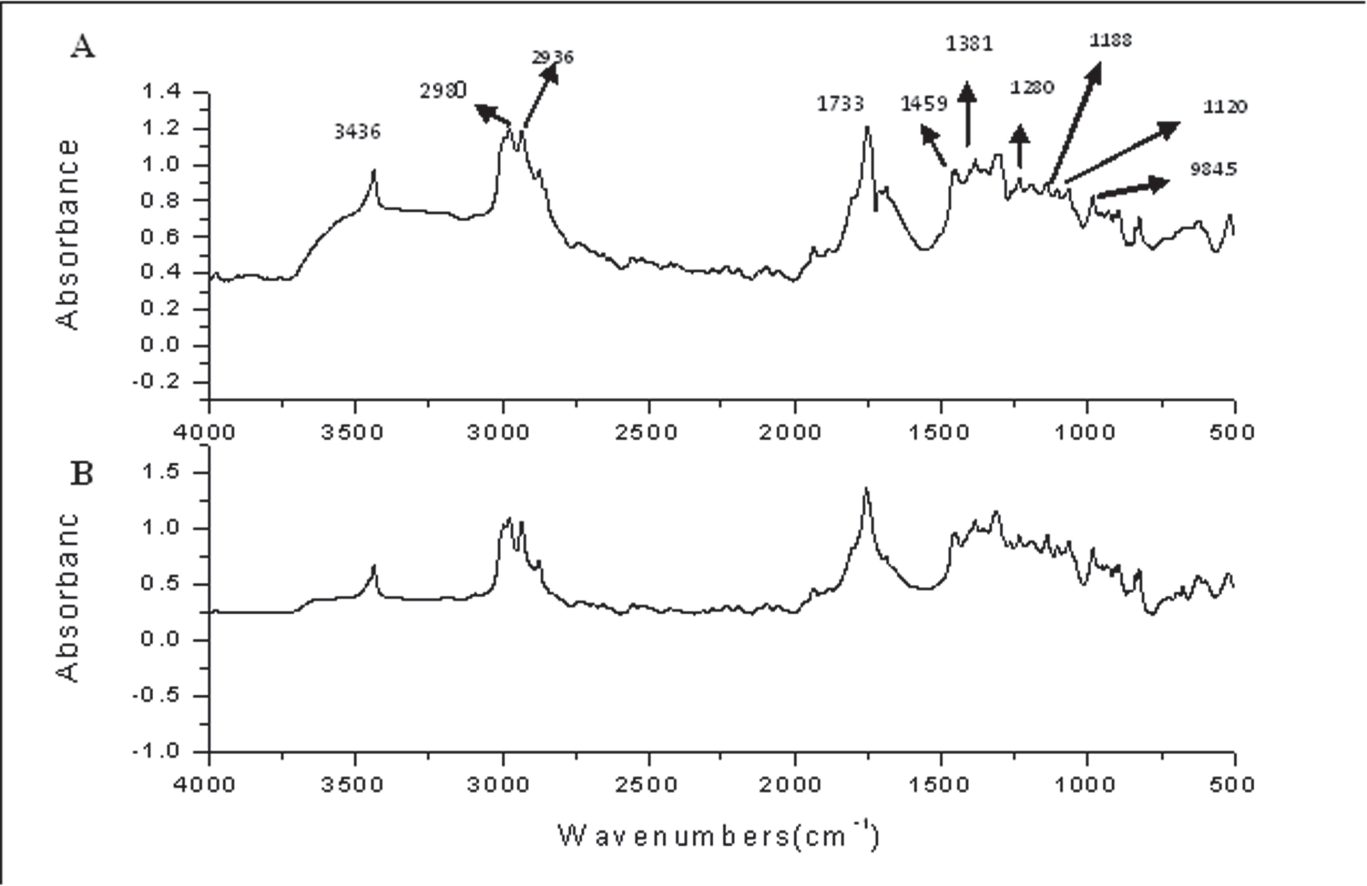
FTIR spectra of standard poly (3-hydroxybutyric acid) (B) and the polymer isolated from the reactor (A).

**5.2 Functional group analysis using FTIR.** PHB crystals were extracted and analyzed through FTIR. The spectrum of the extracted polymer is shown in Fig. 7. The band found at 1459 cm^-1^ corresponded to the asymmetrical C-H bending vibration in the -CH_3_ group while the band found at 1120 cm^-1^ was equivalent to >CH_2_ symmetrical bending vibration. The strong absorption band at 1733 cm^-1^ indicated stretching of the >C = O bond and the band at 1280 cm^-1^ corresponded to the -CH group. The series of bands located in the region of 1000 to 1200 cm^-1^ corresponded to the stretching of the >C-O bond of the ester group. The absorption band around 3000 cm^-1^ was assigned to the terminal -OH group. The absorption peaks obtained are comparable with earlier reports [52, 53] and with the spectrum of pure PHB standard, thus confirming the extracted polymer to be PHB.

FTIR analysis was also used to measure the degree of crystallinity of the polymer. The crystallinity index (CI) is defined as the ratio of the intensity of the bands at 1382 cm^-1^ (CH_3_), which are insensitive to the degree of crystallinity, to that at 1185cm^-1^ (>C-O-C<), sensitive to an amorphous state. CI of the extracted PHB was found to be 0.86, which was lower than a previously reported value of 0.949 [53]. As lower crystallinity induces a faster degradation of the polymer, it is expected to play a key role in determining the polymer stability.

### 6. Microbial community analysis

Total 11252 sequence reads were acquired from 454 sequencing of the microbial community 16S rDNA, after initial filtering 9683 reads were finally selected for further investigation. Detailed results of preprocessing are given in Table 1.

**Table 1:** Quality filters results using RDP pyro sequencing pipeline.

**Initial reads**	**Exponential quality filter**	**Avg. length after trimming**	**S.D. of length**	**Chimeric Sequences**	**Reads after pre processing**
11252	949	384	45.55	620	9683

**Figure 8:**
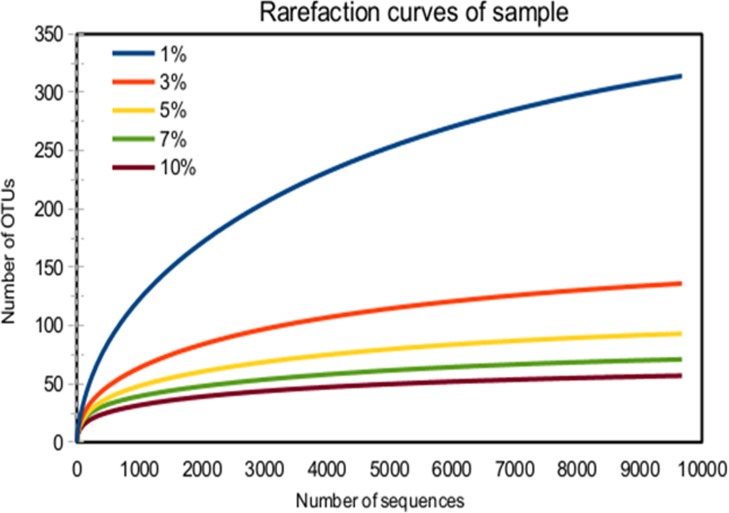
Rarefaction curve of sludge sample at cutoff level of 1%, 3%, 5%, 7% and 10%.

**6.1 Rarefaction curve analysis.** Rarefaction analysis was employed to analyze taxon richness of the sample and to check adequacy of sampling. Plot of the number of sequences against number of OTUs is called rarefaction curve. Distance cut-off values of 0.03, 0.05, 0.07 and 0.10 are generally accepted as points at which differentiation occurs at the species, genus and family/class level, respectively [54]. The upward slope on the curve reflects that a large number of species remain to be discovered while flatter line indicates that almost all the species in the sample have been discovered. It also indicates about the adequacy of the sampling required to discover total species diversity in that ecosystem. Rarefaction curve in Fig. 8 at the cut-off level of 1%, 3%, 5%, 7% and 10% show that slopes tend to be flat, which means the sequence analysis represents almost complete bacterial population and corresponding number of OTUs.

**6.2 Calculation of diversity indexes.** The diversity of sampling was analyzed using Chao1 and Shannon index (H’) (Table 2). “UCI95” and “LCI95” indicate 95% confidence level of upper and lower values of Chao1 for each distance.

**Table 2:** The number of OTUs, Chao1 and Shanon index (H’).

**Level**	**clusters**	**Chao1**	**LCI95**	**UCI95**	**H’**	**Var H**	**E**
1%	314	360.7747	340.7526	395.7816	3.09577	0.00049	0.53845
3%	136	152.24	142.5113	176.5045	2.52768	0.00033	0.51452
5%	93	101	95.47581	118.8502	2.18648	0.00031	0.48239
7%	71	76.5	72.40662	92.50543	2.11933	0.00028	0.49718
10%	57	63.42857	58.51363	84.3029	1.84166	0.00024	0.45551

It can be seen in table 2 that the values of Chao1 index also support the results of rarefaction curve. The evenness “E” refers to how close in numbers each species is in an environment. The H’ value range (1.84–3.09) and species evenness (E = ~0.5) indicate moderate species diversity. This is comprehensible as the current conditions are suitable to augment a specific array of microbial consortia to persistently achieve nutrient removal along with PHB accumulation.

**Figure 9:**
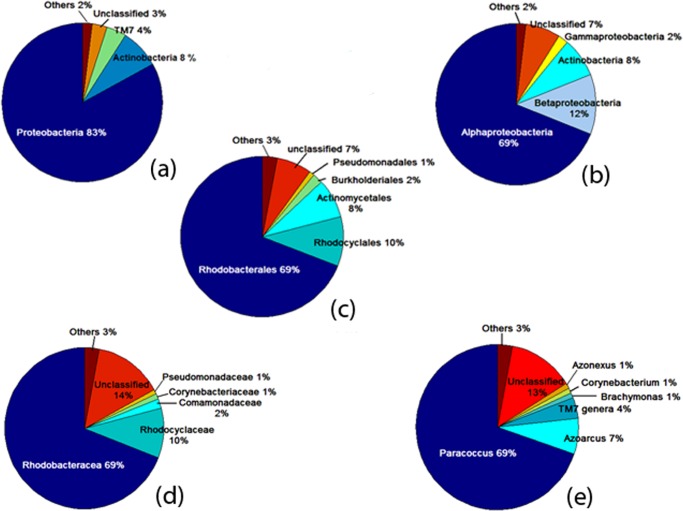
(A) Phylum; (B) Class; (C) Family; (D) Order; (E) Genus.

**6.3 Taxonomic analysis.** Reads were classified into different taxa levels (from phylum to genus) using RDP classifier at 50% thresh-hold as per the recommendation of Cole et al. [24]. The results are highlighted in Fig. 9(A–E).

Details of analysis of the sludge used in the batch experiments have been provided in Tables S1, S2, S3, S4 and S5 in S1 File. When these results were compared with 16S rDNA analysis of sludge from a separate SBR (as mentioned earlier), much similarity was observed in the population dynamics between both the microbial communities (Table S1a, S2a, S3a, S4a and S5a in S1 File) with *Proteobacteria*, *Alaphaproteobacteria*, *Rhodobacterales*, *Rhodobacterececae* and *Paracoccus* being the dominant phylum, class, order, family and genus respectively. These observations further reveal that with change in operational conditions variation in population size were observed in both the sludges, whereas the overall microbial communities were highly similar. The results also show a continuous increase in the unclassified sequences from phylum to family (from 3.53% to 13.61%). It indicates that at the phylum level most of the sequences were classified but at the family and genus level they were not completely categorized. It may also indicate the presence of some novel families or genus of bacteria [54].

Total six bacterial phylum were assigned and *Proteobacteria* (69.23%), *Actinobacteria* (8.35%) and *TM7 (3.53%)* were found to be predominant among them (Fig. 9A).

*Proteobacteria* have been frequently reported to be the most prevailing species in biological waste water treatment plants (WWTP) [54–56]. *Actinobactors* are the filamentous bacteria largely reported in WWTPS performing EBPR [55]. Some also suggest it’s availability in Denitrifying Phosphorous Accumulating processes [57].

Presence of metabolically active TM7 has been observed in activated sludge as well as in WWTPs however their exact function is yet to be elucidated. Researchers are also inquisitive to establish a precise link between TM7and human health as the former has been detected in skin and oral samples of human beings along with its presence in environmental sites [55, 58].

Main classes in the sample were *Alphaproteobacteria* (69%), *Betaproteobacteria* (12%), *Actinobacteria* (8%) and *Gammaproteobacteria* (2%) (Fig. 9B). However this observation was different from previous studies which showed that *Gammaproteobacteria* and *Betaproteobacteria* were predominant in sludge sample [54, 59].

This difference may be attributed to the reactor conditions as it is reported that *Alphaproteobacteria* class performs denitrification and PHB accumulation in anoxic condition under dynamic substrate availability [60] which was further supported by detection of *Paracoccus* (class *Alphaproteobacteria*) as the dominant genus in the sludge sample (Fig. 9E).

12 orders were found in the total bacterial population of the sludge sample. The most dominant orders were *Rhodobacterales* (69.23%), *Rhodocyclales* (9.67%), *Actinomycetales* (8.35%), *Burkholderiales* (1.98%) and *Pseudomonadales* (1.14%) (Fig. 9D).

18 families and 27 genus were detected in the sludge sample. The dominant genus was *Paracoccus* (6695 sequences), *Azoarcus* (684 sequences), *TM7_genera_incertae_sedis* (342 sequences), *Brachymonas* (143 sequences), *Corynebacterium* (140 sequences) and *Azonexus* (138 sequences) which accounted for 69%, 7%, 4%, 1%, 1% and 1% respectively of the total sequence reads. The genus could not be classified for 12.5% sequences. The most prevalent families were *Rhodobacteraceae* (69%), *Rhodocyclaceae* (10%) *Comamonadaceae* (2%), *Corynebacteriaceae* (1%), *Pseudomonadaceae* (1%). Family level classification for 14% sequences could not be done (Fig. 9C).

The most abundant species in this sludge, *Paracocous* (Fig. 9E), is capable of simultaneous phosphate and nitrate removal in wastewater treatment system under anoxic conditions. This species has also been widely reported to uptake short chain fatty acid and store it as polyhydroxybutyrate (PHB) in both WWTP and single culture system [15, 61]. However these microorganisms are unable to utilize PHB for Polyphosphate build up [8, 15] which is also evident in the current study. As the microorganisms tend to show a different behaviour under various growth conditions, therefore the specific mechanism behind substrate utilization and growth is also subject to change under various operational parameters [8, 15, 62–67]. *Azoarocous*, *Azonexus* and *Thaurea*, member of family *Rhodocyclaceae* are reported to be involved in phosphate accumulation [55]. *Corynebacterim* species are well documented as phosphate accumulators in WWTPs [55, 56, 68]. It has been reported that PAOs have the ability to utilize both oxygen and nitrate as electron acceptor [14] therefore in the present case, specific microorganisms might have utilized nitrate as an electron acceptor to accumulate phosphate. Among other sparsely available bacterial population, *Pseudomonas* species have been reported to perform heterotrophic denitrification along with PHB accumulation [69, 70]. *Gammaproteobacteria* widely reported as GAOs [18, 32], *Firmicutes* and other 12.5% of the unclassified bacterial population could be the OHOs/GAOs which attribute to the performance variation of the batch reactors. Though TM7 is one of the most populated groups in the consortium, its exact function is yet to be elucidated.

## Conclusions

Batch studies performed with enriched microbial consortia and varying ICL, under complete anoxic conditions exhibited simultaneous nutrient and COD removal along with PHB accumulation. Within 6 hours of incubation, enriched DNPAOs were capable of achieving maximum COD (87%) removal at 2g/L of ICL whereas maximum nitrate-N (97%) and phosphate (87%) removal along with PHB accumulation (49 mg/L) was achieved at 8 g/L of ICL. Being an anoxic set up, nitrate-N concentration was observed to be the limiting factor as exhaustion of nitrate-N beyond 6 hours of incubation, inhibited COD and the phosphate removal rate which was further revived followed by a fresh supply of nitrate-N to the reaction medium. Adequate nitrate-N availability in the system was therefore found to be a prerequisite to support the continuity of denitrification process under a high carbon load. Presence of nitrate-N in the medium did not hamper PHB accumulation. Further nitrate removal rate was observed to follow a first order kinetics. pH of the reaction medium during the course of the experiment supported the prevailing denitrifying conditions in the medium. Initial carbon, nitrate and phosphate concentrations were therefore found to be interdependent parameters that ultimately determined the fate of the reactor system. A simultaneous high COD/NO_3_ and low phosphate/COD ratio (in ICL-5) was detrimental for nutrient removal as well as PHB accumulation. NMR and FTIR analysis confirmed that the extracted polymer was PHB. 16S rDNA analysis of the microbial consortia of the seed sludge confirmed the dominance of *Corynebacterium, Rhodocyclus*, *Paraccocus solventivorance*, *Paracoccus pantotrophus* and *Paracoccus denitrificans* which are well known for PHB as well as phosphate accumulation using nitrate as an electron acceptor and can provide an economic and sustainable platform for maximum PHB accumulation along with nutrient removal. Rarefaction curve indicated complete bacterial population and corresponding number of OTUs through sequence analysis. Chao1 and Shannon index (H’) was used to study the diversity of sampling and the values of Chao1 index supported the results of rarefaction curve. A continuous increase in the unclassified sequences from phylum to family indicates the presence of some novel families or genus of bacteria in the consortium, contributing to the dynamism of the microbial population in the reactor.

## Supporting Information

S1 File Table S1. RDP classification of sludge samples used in batch studies. S1**a**: RDP classification of sludge samples under changed operational conditions (from another SBR operational under anoxic-aerobic condition). **Table S2.** RDP classification of sludge samples (Class). S2**a**: RDP classification of sludge samples under changed operational conditions (from another SBR operational under anoxic-aerobic condition). **Table S3.** RDP classification of sludge samples (Order). S3**a**: RDP classification of sludge samples under changed operational conditions (from another SBR operational under anoxic-aerobic condition). **Table S4.** RDP classification of sludge samples (Family). S4**a**: RDP classification of sludge samples under changed operational conditions (from another SBR operational under anoxic-aerobic condition). **Table S5.** RDP classification of sludge samples (Genus). S5**a**: RDP classification of sludge samples under changed operational conditions (from another SBR operational under anoxic-aerobic condition).
(DOCX)
