# Supplementary material for: Evaluation of Simultaneous Nutrient and COD Removal with Polyhydroxybutyrate (PHB) Accumulation Using Mixed Microbial Consortia under Anoxic Condition and Their Bioinformatics Analysis
Source: PLoS One. 2015 Feb 17;10(2):e0116230. doi: 10.1371/journal.pone.0116230 (PMC4331290; doi:10.1371/journal.pone.0116230)
Supplement: S1 File — S1a: RDP classification of sludge samples under changed operational conditions (from another SBR operational under anoxic-aerobic condition). Table S2. RDP classification of sludge samples (Class). S2a: RDP classification of sludge samples under changed operational conditions (from another SBR operational under anoxic-aerobic condition). Table S3. RDP classification of sludge samples (Order). S3a: RDP classification of sludge samples under changed operational conditions (from another SBR operational under anoxic-aerobic condition). Table S4. RDP classification of sludge samples (Family). S4a: RDP classification of sludge samples under changed operational conditions (from another SBR operational under anoxic-aerobic condition). Table S5. RDP classification of sludge samples (Genus). S5a: RDP classification of sludge samples under changed operational conditions (from another SBR operational under anoxic-aerobic condition). (DOCX) [file pone.0116230.s001.docx]

**Table S1**: RDP classification of sludge samples used in batch studies

| **Phylotype (phylum**) | **No. of sequences** |
| --- | --- |
| *Proteobacteria* | 8042 |
| *Actinobacteria* | 809 |
| *TM7* | 342 |
| *Chloroflexi* | 87 |
| *Firmicutes* | 54 |
| *Bacteroidetes* | 28 |
| unclassified_phylum | 321 |

**Table S1a**: RDP classification of sludge samples under changed operational conditions (from another SBR operational under anoxic-aerobic condition)

| **Phylotype (phylum)** | **No. of sequences** |
| --- | --- |
| *Proteobacteria* | 7037.00 |
| *TM7* | 1754.00 |
| *Actinobacteria* | 543.00 |
| *Firmicutes* | 349.00 |
| *Chloroflexi* | 57.00 |
| *Bacteroidetes* | 22.00 |
| Unclassified | 23.00 |

**Table S2:** RDP classification of sludge samples (Class)

| **Phylotype (class**) | **No. of sequences** |
| --- | --- |
| *Alphaproteobacteria* | 6704 |
| *Betaproteobacteria* | 1129 |
| *Actinobacteria* | 809 |
| *Gammaproteobacteria* | 187 |

**Table S2a**: RDP classification of sludge samples under changed operational conditions (from another SBR operational under anoxic-aerobic condition)

| **Phylotype (class**) | **No. of sequences** |
| --- | --- |
| *Alphaproteobacteria* | 6480 |
| *Actinobacteria* | 543 |
| *Betaproteobacteria* | 301 |
| *Gammaproteobacteria* | 243 |

**Table S3:** RDP classification of sludge samples (Order)

| **Phylotype (order)** | **No. of sequences** |
| --- | --- |
| *Rhodobacterales* | 6704 |
| *Rhodocyclales* | 936 |
| *Actinomycetales* | 809 |
| *Burkholderiales* | 192 |
| *Pseudomonadales* | 110 |
| unclassified_order | 676 |

**Table S3a**: RDP classification of sludge samples under changed operational conditions (from another SBR operational under anoxic-aerobic condition)

| **Phylotype (order)** | **No. of sequences** |
| --- | --- |
| *Rhodobacterales* | 6479 |
| *Actinomycetales* | 543 |
| *Burkholderiales* | 253 |
| *Xanthomonadales* | 177 |
| *Rhodocyclales* | 47 |
| *Pseudomonadales* | 42 |
| unclassified_order | 1804 |

**Table S4**: RDP classification of sludge samples (Family)

| **Phylotype (family**) | **No. of sequences** |
| --- | --- |
| *Rhodobacteraceae* | 6704 |
| *Rhodocyclaceae* | 936 |
| *Comamonadaceae* | 150 |
| *Corynebacteriaceae* | 140 |
| *Pseudomonadaceae* | 108 |
| *Xanthomonadaceae* | 60 |
| unclassified_family | 1318 |

**Table S4a**: RDP classification of sludge samples under changed operational conditions (from another SBR operational under anoxic-aerobic condition)

| **Phylotype(family)** | **No. of sequences** |
| --- | --- |
| *Rhodobacteraceae* | 6479 |
| *Comamonadaceae* | 244 |
| *Xanthomonadaceae* | 176 |
| *Corynebacteriaceae* | 109 |
| *Rhodocyclaceae* | 47 |
| *Pseudomonadaceae* | 26 |
| Unclassified | 2195 |

**Table S5**: RDP classification of sludge samples (Genus)

| **Phylotype (genus)** | **No. of sequences** |
| --- | --- |
| *Paracoccus* | 6695 |
| *Azoarcus* | 684 |
| *TM7_genera_incertae_sedis* | 342 |
| *Brachymonas* | 143 |
| *Corynebacterium* | 140 |
| *Azonexus* | 138 |
| unclassified_genus | 1217.00 |

**Table S5a**: RDP classification of sludge samples under changed operational conditions (from another SBR operational under anoxic-aerobic condition)

| **Phylotype (genus)** | **No. of sequences** |
| --- | --- |
| *Paracoccus* | 6473 |
| *TM7_genera_incertae_sedis* | 1754 |
| *Brachymonas* | 218 |
| *Corynebacterium* | 108 |
| *Azoarcus* | 32 |
| *Azonexus* | 6 |
| Unclassified | 726 |
